# A Qualitative Exploration of the Meaning and Understanding of Male Partner Involvement in Pregnancy-Related Care among men in rural South Africa

**Published:** 2017

**Authors:** Motlagabo G. Matseke, Robert A. C. Ruiter, Nicole Barylski, Violeta J. Rodriguez, Deborah L. Jones, Stephen M. Weiss, Karl Peltzer, Geoffrey Setswe, Sibusiso Sifunda

**Affiliations:** Human Sciences Research Council, South Africa, and Maastricht University; Maastricht University; University of Miami Miller School of Medicine; University of Miami Miller School of Medicine; University of Miami Miller School of Medicine; University of Miami Miller School of Medicine; Mahidol University and University of Limpopo; University of Venda; Human Sciences Research Council, South Africa

**Keywords:** male partner involvement, pregnancy-related care, male perceptions, South Africa

## Abstract

Male partner involvement (MPI) during antenatal care has been promoted as an effective intervention to improve maternal and newborn health outcomes. Although MPI is commonly defined as men attending antenatal clinic visits with their female partner, few men attend antenatal clinic visits in rural communities in the province of Mpumalanga, South Africa. The study aimed to qualitatively explore the meaning and understanding of MPI as perceived by men visiting primary health care clinics in rural communities in Mpumalanga. Six focus groups discussions (*n* = 53) were conducted, digitally recorded, simultaneously transcribed, and translated verbatim into English. Data were analyzed using thematic content analysis. Perceptions of male roles during and after pregnancy differed among men. Male involvement was understood as giving instrumental support to female partners through financial help, helping out with physical tasks, and providing emotional support. Accompanying female partners to the clinic was also viewed as partner support, including behaviors such as holding a spot for her in the clinic queues. Community attitudes, traditional beliefs, and negative experiences in health facilities were barriers for MPI. This study provides support for concerted efforts to work with both men and women within the cultural context to explore the important roles of all members of the family in working together to provide the best possible health outcomes for mother and infant. In particular, future interventions should focus on making antenatal care services more responsive to male partners, and improving male partner accessibility in health care facilities.

## Introduction

Developing countries account for about 99% of global maternal deaths (World Health Organization [WHO], 2014). In many such countries, men are the key decision makers and chief providers, often determining women's access to economic resources, and may greatly influence behavior regarding the use of contraceptives, availability of nutritious food, women's workload; and the allocation of money, transport, and time for women to attend health services (Nesane, Maputle, & Shilubane, 2016; Yargawa & Leornardi-Bee, 2015). In all of these ways, men can play a central role that greatly influences maternal and infant health outcomes.

Pregnancy necessitates a number of critical decisions, such as attending clinic appointments, choosing the delivery method, and determining how to feed the infant—all decisions that can be greatly enhanced by the involvement of the father of the baby or the male partner. Traditionally, maternal health issues including pregnancy and childbirth have predominantly been seen and treated as feminine matters (Kinanee & Ezekiel-Hart, 2011; Singh, Lample, & Earnest, 2014), making male partner involvement (MPI) in these issues not a priority. However, many men, when given an opportunity, are willing to be positively involved in matters affecting reproductive health (Levtov, van der Gaag, Greene, Kaufman, & Barker, 2015; van den Berg et al., 2015). Studies indicate men's willingness to be involved in the care of their female partners during pregnancy, at birth, and after birth (Bhatta, 2013; Daumbaugh et al., 2014; Kaye et al., 2014; Vermeulen et al., 2016). In a study by Kaye et al. (2014) in Uganda among men who came to the hospital to visit their admitted partner, most men were willing to learn about their expected roles before and during childbirth and were eager to support their partners during this time.

Male partner involvement during and following pregnancy has been promoted as an effective intervention to improve maternal and newborn health outcomes. There is evidence that MPI promotes better maternal health outcomes and improved health behavior. A systematic review of 14 studies on MPI and maternal health outcomes indicated that MPI during pregnancy significantly decreased the likelihood of antenatal and postpartum depression, decreased likelihood of childbirth complications, and improved utilization of maternal health services such as skilled birth attendance (Yargawa & Leornardi-Bee, 2015). Comparable results were found in a study conducted by Kaye et al. (2014), where it was reported that MPI reduces maternal stress through emotional, logistical, and financial support (Yargawa & Leornardi-Bee, 2015). In South Africa, lack of MPI was associated with depressive symptoms among a sample of HIV positive pregnant women (Peltzer, Rodriguez, & Jones, 2016). Male partner involvement has also been associated with increased adherence to antiretroviral medication during pregnancy (Peltzer & Shikwane, 2011).

The meaning and understanding of MPI during and following pregnancy may vary in different contexts, but it is most commonly defined as a man's physical presence in the antenatal or postnatal clinic with his female partner (Montgomery, van der Straten, & Torjesen, 2011). Furthermore, MPI is defined as men taking part in their pregnant partner's birth plans, encouraging exclusive breastfeeding and immunization for their children; supporting their partners, and communicating about pregnancy-related health care (Bhatta, 2013; Montgomery et al., 2011). Men can support their partners by helping to prepare for delivery, saving money, arranging transportation to the birthing center, reducing workload during pregnancy, and providing emotional support (Bhatta, 2013; Vermeulen et al., 2016).

Studies in Africa and elsewhere have highlighted challenges of MPI in pregnancy-related health care services. Barriers to MPI in antenatal health care services have been identified as personal, family, community, and health systems factors. Studies have cited the following personal- and family-level factors as barriers to MPI in antenatal care and related services: unwillingness of men to know their HIV status (Aarnio, Olsson, Chimbiri, & Kulmala, 2009), men's lack of knowledge about the services (Nyasulu, 2007), marriage instability (Aarnio et al., 2009), fear of potential dissolution of marriage consequent to disclosure of HIV-positive status, fear of stigmatization, and lack of appropriate communication between partners (Kanyama et al., 2004). Community-level factors include culturally embedded traditional gender norms that discourage men from attending health services (Kura, Vince, & Crouch-Chivers, 2013; van den Berg et al., 2015).

Most countries in Sub-Saharan Africa are patriarchal in nature, wherein men perceive antenatal care to be the sole responsibility of women and practice gender norms that disapprove of males engaging in antenatal care activities (Auvinen, Kylmä, & Suominen, 2013; Dunlap et al., 2014; Farquhar et al., 2004; Morfaw et al., 2013). Women, in turn, perceive men's primary role to be financial, specifically to pay for antenatal care services (Dunlap et al., 2014). Men who had fathered a child and were no longer involved, or were *absent fathers*, admitted that poverty also prevented them from being involved, because they identified the role of father with the role of being a financial provider (Eddy, Thomson-de Boor & Mphaka, 2013). Health system barriers include health delivery system factors (Chinkonde, Sundby & Martinson, 2009), such as services offered in an area traditionally viewed as a woman's domain (Aarnio et al., 2009). Men have found the health care settings to be unwelcoming, intimidating, and unsupportive (Kaye et al., 2014).

There is a widely held belief that MPI is very important (Singh et al., 2014; WHO, 2015; Yargawa & Leornardi-Bee, 2015), yet there is no common understanding of what MPI is, and its meaning may vary in different contexts. Male partner involvement has been highlighted as a maternal, newborn, and child health priority in South Africa and other countries and, as such, has been promoted as an effective intervention to improve maternal and newborn health outcomes (Joint United Nations Programme on HIV/AIDS, 2011; Levtov et al., 2015; South African National AIDS Council, 2011; WHO, Department of Reproductive Health and Research, 2012). The province of Mpumalanga, South Africa, is predominantly rural and is characterized as one of the areas with the highest maternal mortality ratios and neonatal death rates in South Africa (i.e., 115.4/100,000 and 7.9/1,000 births, respectively, in 2014 and 2015; Mpumalanga Province Health Department [MpuDoH], 2016). Nkangala District had the highest maternal mortality ratio in the province at 198.1/100,000 births, which is much higher than the provincial average. Gert Sibande District had the highest neonatal death rate in the province at 8.9/1000 births, which is much higher than the provincial average. The provincial average rate of antenatal visits before 20 weeks is low at 55.5%, with both the Gert Sibande and Nkangala districts having even lower rates, at 46.7% and 54.5%, respectively (MpuDoH, 2016).

Despite the importance attached to men's involvement in South Africa, there is limited research on MPI during and following pregnancy, particularly from males' perspectives, which impedes the development of contextualized appropriate interventions. The main aim of this article is to explore the meaning and understanding of MPI among South African men and to consider strategies for culturally appropriate integration of male partners in antenatal and postnatal care programs in rural clinics in Mpumalanga.

## Theoretical Framework

Male partner involvement in pregnancy-related care in the present study was informed through using social support theory. Social support is a positive social interaction and is described as the help provided through social relationships and interactions (Bartholomew Eldredge, Parcel, Kok, & Gottlieb, 2011). The four main types of social support are emotional (provision of empathy, love, trust, and caring), instrumental (provision of tangible aid and services), informational (provision of advice, suggestions and information), and appraisal (provision of feedback useful for self-reevaluation and affirmation; Bartholomew Eldredge et al., 2011).

Male partner involvement can take the form of a positive social interaction between two partners in an intimate relationship who, together, need to make efforts and important decisions for the health of the expected baby. A male partner can provide instrumental or emotional support to his pregnant partner who needs antenatal and postnatal care services. In showing support for his female partner, a male partner can encourage her to attend (and accompany her to) antenatal care, help prepare and save money for delivery and arrange transportation to the birthing center, support good nutrition, reduce workload during pregnancy, and provide emotional support (Bhatta, 2013; Vermeulen et al., 2016).

The meaning attached to MPI and men's understanding of MPI in antenatal and postnatal care may be explained by their attitudes toward it, their subjective norms, and perceived behavioral control regarding MPI. The theory of planned behavior (TPB) was used as a guide to explain men's willingness with regard to supporting their pregnant partners during and after pregnancy. The TPB suggests that intention, the most important determinant of behavior, is determined by three conceptually independent constructs: attitude, subjective norms, and perceived behavioral control (Ajzen, 1988). According to TPB, attitudes toward a certain behavior (MPI, in this case) are influenced by beliefs about what is entailed in performing the behavior and outcomes of the behavior (Glanz & Rimer, 1997). Subjective norms are influenced by beliefs about social standards and motivation to comply with those norms (Glanz & Rimer, 1997). Perceived behavioral control is affected by the presence or lack of things that will make it easier or harder to perform the behavior (Glanz & Rimer, 1997).

## Method

An exploratory qualitative study was conducted between August 2015 and June 2016 at six selected primary health care clinics in Mpumalanga. Six focus group discussions (FGDs) were conducted and consisted of 53 men in total from communities representing both periurban and rural areas. Clinics were purposively selected from each of the five subdistricts represented in the study (i.e., Thembisile Hani, Msukalikwa, Dipaleseng, Emalahleni, and Govan Mbeki).

### Study Setting

The study was conducted at six primary health care clinics situated in Nkangala District (with a population of 1,407,465) and Gert Sibande District (with a population of 1,076,612), in Mpumalanga (MpuDoH, 2016).

### Selection and Recruitment of Participants

Male participants were recruited using convenience sampling by study fieldworkers based at the clinics, with assistance from clinic staff. Men who were visiting the clinic were referred to the study fieldworkers by clinic nurses after receiving health service. The fieldworkers then explained the study to each of the men; those who were willing to participate in the study were taken through an informed consent process. Men were then scheduled for a group session on a specific date and time at the clinic. A list was compiled of all men who were going to be part of a FGD in each clinic. Each men's FGD consisted of about eight to 10 participants.

The inclusion criteria for men were as follows: (a) Participants must be men who have fathered at least one child in their lifetime or have a partner who is currently pregnant (it was assumed that all participants would have been exposed to antenatal care services by having a pregnant wife and would thus be able to make a significant contribution during the discussions), (b) participants had to be willing to participate in the FGDs, (c) participants had to be able to give written consent, and (d) participants had to be 18 years of age or older.

### Data Collection

Focus Group Discussions were conducted by study researchers in either seSotho or isiZulu, took about 45 minutes on average, and were audio-recorded. Participants were each compensated South African Rand 50 (∼US$5) for their participation.

### Focus Group Guide

The FGD guide was developed based on literature review from similar studies in Sub-Saharan Africa (Brittain, 2014; Mohlala et al., 2012). Questions were derived from FGDs that had been conducted with pregnant women who were part of a larger study in community health centres in two districts in Mpumalanga (Jones et al., 2014). The questions were specifically aimed at eliciting the understanding of MPI in the South African socioeconomic and cultural context. The study personnel followed a FGD guide, and all held a masters- or doctoral-level degree.

The following questions were used as guides to introduce topics for the discussions:

- What is the understanding and interpretation of MPI in child care before the baby is born?
- In what ways should male partners be involved in supporting their partners while they are still pregnant?
- Should men accompany their partners to clinic appointments during pregnancy, and why?
- What could be some of the reasons that would make it difficult for men to accompany their partners when they go to the clinic during pregnancy?
- What kind of experiences have men had when attending clinic appointments with their partners?
- What are the attitudes of clinic staff toward males attending clinic appointments with their partners?
- What is the understanding of MPI during the birth of the child?
- What is the understanding and interpretation of MPI in child care after the baby is born?
- How does the general community define or understand the meaning of MPI during and following pregnancy?

### Data Analysis

The audio-recorded FGDs were simultaneously transcribed and translated verbatim into English by project staff who took notes during the discussions. These notes were used as a basis for the transcripts. Grounded theory (Glaser & Strauss, 2009) was used for coding and analyzing of transcripts line by line. The analysis focused on understanding different aspects of MPI among participants. Open, axial, selective, and theoretical coding strategies were employed (Glaser, 2005). Thematic disagreements, though rare (<5%), were discussed until consensus was reached among coders. Furthermore, discussions were conducted to redefine codes and themes. Coders also considered how knowledge, assumptions and beliefs may have influenced codes, themes, and the overall qualitative analytic strategy. Finally, coders and authors used theoretical memoing (Glaser, 1998) to compare and contrast different domains of MPI pre- and postnatally.

### Ethical Considerations

Prior to study onset, approval was obtained from the Human Sciences Research Council Research Ethics Committee, the University of Miami Institutional Review Board, and the Mpumalanga Department of Health and Welfare (provincial, district, sub-district and clinic levels). Written informed consent for participation and digital recording was obtained from each participant.

## Results

Participants (*n* = 53) had an average age of 35.5 years (range between 26 and 50 years). Seven themes were identified and included: perception of male roles, prenatal MPI, postnatal MPI, men's willingness to become involved, challenges at clinic level, community attitudes and beliefs, and challenges at family and partner level.

### Perception of Male Roles

There were different perceptions of male roles in supporting their partners before and after birth. Both instrumental and emotional support, as explained in the social support theory, clearly describe men's perceptions of their roles in supporting their female partners. All participants believed that a man's role was to care for and provide for his significant other, but they disagreed on the extent to which, and manner in which, this should be done. While some men thought that expressing love and helping out with some physical tasks for their partner were necessary, many men thought that providing financially was the primary duty in supporting their partners. One participant said, “To give her love and take care of her”.

Those who felt that only financial contributions were required were of the opinion that it is a cultural norm and an expectation for men to provide financially for their families and not do any physical tasks to support their pregnant partners:

[T]he society we are living in believes that a man can only be involved financially. As men, they think that you must only provide financially and even the elders will tell the exact same thing. Bonding with your child and things like that, they only believe it's for women to do and men become pressurized.

As explained by the TPB, men's perceived role of being a financial provider was influenced by their traditional societal beliefs and norms and their motivation to comply with those norms. Traditionally, in most ethnic groups in South Africa, men were seen as (financial) providers, and it would seem culturally inappropriate to involve men in the caring duties in households. It seems some men in the FGDs felt that they are under pressure to perform womanly duties if they support their partners in any way other than financially.

### Prenatal Male Partner Involvement

Men had varying perceptions of their roles during the prenatal period. All the men agreed that their partners should be given support during pregnancy. Some advocated for increased sharing of the workload, others for avoiding emotionally wounding her, and still others advocated for simply bringing her to the clinic and waiting in line but not attending the actual antenatal care appointment. One participant was quoted as saying,

Yes, I know that it's always full here at the clinic but I'm not staying far from the clinic so I would wake up at 5 o'clock in the morning to stand the queue and my partner would come around 7 and by that time I'd be number 2 or 3. When she gets in I would go back home to drink tea or do whatever that I want to do and she'll be back home around 9.

A few men showed support for attending the actual antenatal care visit with partner. Those who mentioned that they had attended clinic visits with their pregnant partner did it so that they could have access to the same information as their partner. However, it seems as if sometimes a man could not attend the antenatal care appointment due to restricted access. One participant put it this way:

I used to accompany her to the clinic always, they'd check her and give her other dates and by doing that I was also aware of her dates. Sometimes they'd agree but sometimes I was not allowed to go in.

### Postnatal Male Partner Involvement

Men had mixed views regarding ways in which their partner could be supported following pregnancy. While some men believed that only providing money was necessary, others believed that they should provide other forms of instrumental support. Some participants spoke of taking more of a backseat role because of a lack of knowledge of paternity leave or an unwillingness to miss work to care for the baby. However, even when taking the backseat, some agreed that men should provide financial support. Others felt that their culture dictated that they remain distant in the early days of the baby's life:

You can't just go around and come back to hold your newborn baby. After the baby is born, even after work, you can't just go in where the baby is. You should keep your distance, and during that time you must sleep in the visitor's room until the baby is 10 days old.

It seems one participant understood that taking an active role in caring for the baby, like bathing him or her, would be good, as this will make the baby feel comfortable with both parents:

Bathing the baby is our responsibility, and the baby must know that these people are both my parents. Also, if the baby needs something, he or she can go to either of us, even if the baby wants to bathe he'll be able to go to both of us.

Other participants still believed that they should continue supporting their partners by taking over some of the household workload, even after birth:

I believe that you need to take care of the house chores, like cleaning, cooking and so on. You'll find that some of the women give birth through C-section and she won't be able to bend and do the chores, so you need to take over those departments and you also need to do the washing.

### Men's Willingness to Be Involved

This aspect is explained by the TPB in that some men's willingness to be involved seems to have been determined by their attitudes toward MPI and their perceptions of whether their significant others approve or disapprove (subjective norm) of MPI. The majority of men showed unwillingness and embarrassment (negative attitude) in taking time off/leave from work to allow time to care for their child. They believed that the task of caring for the baby and showing related support to the partner should be left to women (subjective norm) and other trained professionals. One said, “Men don't like to take leave.” Another said,

I think trained people are the ones who are allowed in there, and there are also male nurses who are trained to do the job; only if you're trained you can do the job but if you're not trained you can't do the job.

Some men reported that when they did take time off, they often spent it doing things that gave them pleasure, such as drinking beer, rather than using this time to give support to their partner. One participant was quoted as saying,

It's just we, as men, are ignorant, and we don't take things seriously and also we have fear, that's why men are seen as failures. I can take those three days, only to find out that I took those three days to do my own things.

A few spoke of standing firm against this culture and caring for their child and being involved, regardless of what others would think (positive attitude and perceived behavioral control).

### Challenges at Clinic Level

Participants shared their experiences when attending clinic appointments with their partners and commented on staff attitudes toward men. Overall, it seemed that men found the clinics unfriendly and intimidating. Participants mentioned that the majority of the people attending clinics were women, so any men who attended felt out of place. Additionally, long clinic waiting times discouraged men from attending, as they were often impatient and had other things to do, such as getting back to their workplaces: “What I've noticed is that we don't have patience, and another thing that makes us impatient is the long queues that we have to stand in at the clinic, and also we have busy schedules during the day.” One participant shared a positive experience when he visited his partner the day after their baby was born—he was praised and encouraged by women at the ward when he held his baby despite being the only man there:

I was the only one who was holding my baby and I felt like I was betraying men, so that's why I decided to return there again. But it was for a good cause, because many women were praising me and encouraging me, saying I'm doing a good thing.

In some clinics, participants mentioned that clinic staff did not allow men into certain areas, such as the labor ward, and it was not clear to men in which areas they were allowed to go. This limited their involvement in the process:

The issue is, there should be a male section here at the clinic, because I got lost once and I found myself in the in the maternity ward. I was told that men are not allowed in there, so it would be nice to have a section were women are not allowed, but men, and that would encourage us to come to clinic.

Unwelcoming staff attitudes, inconsistent schedules, and stringent rules regarding male attendance in the clinics also appeared to discourage men from attending. It was suggested that creating areas specifically for men would encourage them to be more involved. This is what one participant had to say:

I can't remember well, but it was for six-months or six-weeks appointment, the way the sisters were talking to me made me think twice about coming back again, because they'd say, “Ladies please shift to the other side.” By that time, I'd be among them so it made feel that they don't even care that I'm also there too for my baby's appointment.

In addition, some men felt that clinic space could not accommodate men. In some cases, they were not allowed to be present during the birth of their child because multiple women were already in the room. The lack of privacy also seemed to affect men's ability to communicate and express themselves. With multiple patients being simultaneously attended to, it seemed staff did not have the opportunity to explain procedures to men. One participant reported feeling lost during the process despite being allowed to participate: “During my wife's pregnancy, they didn't allow me to get in, because there were so many pregnant women in that room and you'd find they see three or four pregnant women at the same time.” Another said,

Yes sometimes we feel ashamed because you're in queue of pregnant ladies and yet you don't know how it feels to be pregnant. As you know, that makes it difficult for men to express themselves among women. We just follow the procedure but sometimes we don't know what's going on.

### Community Attitudes and Beliefs

Community views of MPI in pregnancy-related matters were discussed by participants. They also discussed reasons it was difficult to accompany their partners to antenatal and postnatal care visits. Drawing from the TPB, men's behavior (supporting their female partners) was determined by their subjective norms, which are influenced by their perceptions regarding traditional societal beliefs and norms in prenatal issues. Participants reported that the clinic environment and pregnancy-related matters were considered by their communities to be in the women's domain: “In our Black culture, there are things that should be done by women and not by men but now when you accompany your wife to the clinic people might think otherwise, and they'll gossip behind your back.”

Men who attended clinics with partners felt uncomfortable; some mentioned that they would be judged or deemed *bewitched* if they attended the clinics with their partners. Many feared that if they assisted in the housework or care of the baby, it would appear that their wives had given them *isidliso* (meaning a love potion): “For an example if your wife is at work during the week and… then you decide to do the washing during the weekend, some people would think that your wife has given the love potion.” According to the men, they would be stigmatized if others found that they were involved in caring for their partner during pregnancy. This is what one participant had to say about when a man accompanies his partner to the clinic: “Yes, in our Black community ‘isidliso’ makes a man to be a laughing stock and they'd think you're not mentally fit.”

### Challenges at Family/Partner Level

Family- and partner-related issues that limited male involvement were raised by participants. According to participants, some women were reluctant to have men present for the actual birth of their baby, because the women feared being teased for their actions when they were giving birth or after delivery. Participants also felt that women feared that having men present might negatively affect their male partner's psyche. One man shared that he might not be ready to witness childbirth, as it would involve seeing his partner screaming:

I think some women, they don't like us to see the actual birth.

I haven't seen that, but I can imagine, you as man, it will affect you in a certain way.

I don't think I would stand the screams, when she screams I would also end up crying.

Another issue was that during the late stages of pregnancy, a woman is often encouraged to go live with her family, causing her partner to feel disconnected and isolated from the pregnancy/birthing process. Some men even felt that women withheld information from them. One participant felt that despite a man's desire to ensure his child's safety by being more involved and learning more about caretaking, the woman would conceal information from him. Another shared that women may not want men to be involved in their child's life and may go as far as to prohibit men from seeing the infant:

Sometime(s), they spend 30 days at their parent's home after giving birth. But it becomes a challenge, because you must go there every day to visit the baby, and that's where they think you might bring evil spirits. In some cases, before you can enter the room where the baby is, they first burn the incense. Even my mother told me, that if you have been having sex a night before you visit your baby, the baby would have a foam-like thing coming from the mouth.

## Discussion

The present study examined the meaning and understanding of MPI among men visiting primary health care clinics in rural communities in Mpumalanga. Men's understanding of their roles as perceived by the community, within the cultural context, and the health care setting, highlighted important focal points to consider in the planning of interventions aimed at improving MPI during and following pregnancy in rural South Africa.

Perceptions of MPI differed among men; however, they were generally understood as giving partners instrumental support through running errands, sharing domestic chores, and providing financial and emotional support. Men who defined partner support as limited to giving financial help did not endorse the view of carrying out physical tasks for pregnant women, as they mentioned that these tasks were meant for women according to the *elders* and the *society* in which they resided. These findings are similar to those in the Singh et al. (2014) study, in which men from two villages in Uganda believed that issues related to pregnancy and childbirth were in the women's domain, with men's main responsibility being provision of funds. Evidently, there is a need for a community-level intervention aimed at the normalization of MPI during and following pregnancy. For example, an intervention was implemented in the community of Ekiadolor in Nigeria, where group health talks were conducted, applying local culture and gender norms among males to improve their attitudes and practices regarding their involvement in prenatal care (Adeleye, Aldoory, & Parakoyi, 2011).

Accompanying partners to the clinic was also perceived to be a form of partner support by most men, even though they did not necessarily attend the antenatal care appointment. Holding a spot for women in the waiting queues or making arrangements for transportation to and from the clinic were considered equivalent to participation in the clinic visit. This is in contrast to the current literature, which commonly defines MPI as attending the clinic visit with the female partner (Alusio et al., 2011; Bhatta, 2013; Montgomery et al., 2011).

Culture and community structures play a role in men's perceptions of MPI, subsequently influencing their behavior. Hence, men expressed anxiety about the opinions of community members regarding support of their partners. Some men responded to this perceived social norm by choosing to limit support and to avoid being seen as *bedeviled* by the woman. In other instances, men felt it was not possible to be involved, either due to their partner's personal issues or because partners choose to distance themselves from the men during late stages of pregnancy upon observing such cultural norms. Men's perceptions and attitudes toward MPI, including those of the community, need to be improved if desired male involvement is to be achieved. This can be accomplished through the implementation of evidence-based interventions. The Men as Partners program in South Africa, for example, manipulated gender norms ascribed to traditional partner relations and challenged male attitudes and behaviors that compromised the health of women (Peacock & Levack, 2004). The program, as explained by Peacock and Levack (2004) was carried out through a partnership of civil society organizations collaborating with governmental and academic institutions and sought to transform men's attitudes and behaviours to promote their constructive role in sexual and reproductive health, including HIV/AIDS.

Negative experiences in health care facilities were also mentioned as barriers to MPI. Men in this study felt the clinics were unfriendly. They reported unwelcoming clinic staff attitudes, felt that the clinic space could not accommodate them, and noted stringent rules regarding male presence in some clinic spaces. Similar results were reported in other countries with health care delivery systems that fail to welcome men and further limit men's participation to culturally defined traditional gender roles (Auvinen et al., 2013; Chikonde et al., 2009; Dunlap et al., 2014; Morfaw et al., 2013; Tadesse, Muula, & Misiri, 2004). Future interventions should focus on making health care facilities to be male friendly and antenatal care services more responsive and acceptable to male partners. This can be achieved, for example, by engaging maternal, neonatal and child health or antenatal care staff as agents in transforming facilities into gender-sensitive spaces, where women and men are welcomed as clients (van den Berg et al., 2015). Furthermore, staff members in health care facilities need to be equipped to recognize and sensitively discuss with clients how gender-inequitable beliefs and behaviors adversely affect health.

The current study sought to identify and clarify MPI during and following pregnancy within the South African context. The men in this study established their own vision for providing support for their partners. However, it is not clear that the outcomes attributed to MPI are clearly linked to women's health outcomes, and results suggest the importance of further research on the role of MPI in South Africa.

This study has limitations inherent in qualitative research: The opinions of a sample of men may not necessarily be generalizable to all population groups in South Africa. Additionally, the majority of men lived in rural settings, where traditional roles may be more dominant than elsewhere in South Africa, and the activities identified as representing MPI do not encompass all possible roles. Previous research has characterized MPI as clinic attendance, partner support, communication, knowledge of HIV and prevention of mother-to-child transmission of HIV, and disclosure of HIV status with the purpose of promoting adherence to prevention of mother-to-child transmission practices, and to include accompanying partners to clinic visits (Jennings et al., 2014; Montgomery et al., 2011).

Challenges to MPI identified in this study have been previously addressed and included personal, family, community, and health systems factors (Nyondo, Chimwaza, & Muula, 2014). Future initiatives should address known barriers to MPI, including concerns about HIV testing and status disclosure (Aarnio et al., 2009; Nyasulu, 2007; Nyasulu & Nyasulu, 2011), poor communication between partners, and fear of stigmatization (Kanyama et al., 2004).

## Conclusion

The present study provides support for concerted efforts to work with both men and women within the South African context, to explore the important roles of all members of the family, in working together to provide the best possible health outcomes for mother and infant. In particular, future interventions should focus on making antenatal care services more responsive to male partners and improving male partner accessibility in health care facilities. Cultural level challenges, which may limit men's willingness to become involved in pregnancy related care, may be surmounted as social norms change.
